# Buffered poincaré plot: a robust visualization method for improving automatic atrial fibrillation diagnosis

**DOI:** 10.1093/bib/bbaf631.025

**Published:** 2025-12-12

**Authors:** Masanao Yasuoka, Hironori Shigeta, Tomoaki Nakano, Keita Okayama, Shigeto Seno

**Affiliations:** The University of Osaka; The University of Osaka; The University of Osaka; The University of Osaka; The University of Osaka

## Abstract

Atrial fibrillation (AF) is a common arrhythmia that may lead to severe outcomes, including heart failure and cerebral infarction. AF diagnosis is commonly based on electrocardiograms (ECG), and traditionally requires experts to visually inspect long-term recordings, which is time-consuming and labor-intensive. Therefore, the development of automatic AF detection technologies is essential to improve diagnostic efficiency and accuracy in clinical practice.

Recently developed wearable Holter ECG devices, integrated into clothing, enable continuous daily ECG recording. However, ECG data from these devices often include numerous segments with measurement failures. The Poincaré plot is a tool to visualize nonlinear features and rhythm complexity of heart rate variability, and has been utilized for automatic AF detection. However, erroneous RR-intervals (RRI) arising from failure segments can distort both visualization and quantitative assessment in conventional Poincaré plots.

In this study, we propose the Buffered Poincaré plot, which selectively visualizes diagnostically relevant RRI while filtering out failure regions in wearable Holter ECG data. The proposed method evaluates the consistency of each RRI with previously observed signals, retaining only physiologically consistent intervals and filtering out noise-like sequences. The accuracy of automatic diagnosis using statistical metrics in Buffered Poincaré plot improved by approximately 8% compared with conventional methods.

